# Psychology students' attitudes towards research: the role of critical thinking, epistemic orientation, and satisfaction with research courses

**DOI:** 10.1016/j.heliyon.2021.e08504

**Published:** 2021-12-01

**Authors:** Miguel Landa-Blanco, Antonio Cortés-Ramos

**Affiliations:** a Universidad Internacional Iberoamericana, Mexico; b Universidad Nacional Autónoma de Honduras, Honduras; c Universidad de Málaga, Spain

**Keywords:** Scientific attitudes, Critical thinking, Epistemology, Student research

## Abstract

The current study aimed to determine how attitudes towards research are related to epistemic orientation, critical thinking, and satisfaction with research courses in psychology university students. Control variables included respondents' gender, current academic degree (undergraduate or postgraduate), number of research methods courses completed, number of research projects completed, and academic score. A quantitative, cross-sectional design was used, with a non-probabilistic sample size of 137 students. Correlational findings suggest that students with high scores in critical thinking domains and empiric and rational dispositions, tend to achieve higher academic grades. Rationality and reflexive skepticism were related to the number of research projects completed by the student. While an intuitive disposition is inversely related to academic scores and the number of research courses completed. Results from a hierarchical linear regression model suggest that attitudes towards research are significantly and positively affected by students' satisfaction with research courses, empiric epistemic orientation, and critical openness. On the other hand, an intuitive epistemic orientation has significant detrimental effects on attitudes towards research. Rational epistemic orientation and skeptic reflexiveness yielded non-significant coefficients. Overall, the model containing all independent variables accounted for 47.4% of the variance in attitudinal scores; this constitutes a large effect size. Results are discussed in light of previous research and their implications for the teaching of psychology in higher education.

## 1 Introduction

Attitudes are defined as a cognitive preference and behavioral predisposition towards an object, thus resulting in a favorable or unfavorable evaluation regarding a specific stimulus (Eagly and Chaiken, 1993). Attitudes play an important role in predicting behavior (Glasman and Albarracín, 2006), and consequently are a recurrent topic in educational and psychological studies. The present article will focus specifically on psychology students' attitudes towards research.

Research skills play an important role in higher education (Lambie et al., 2014) and the psychological sciences (Veilleux and Chapman, 2017). In higher education, specific competencies within psychology include the epistemic comprehension of science, critical scientific thinking, as well as the capability to design, execute and understand research (American Psychological Association, 2011). However, on many occasions, psychology students dislike research methods courses (Ciarocco et al., 2012). This might be due to the fact that students perceived disconnection between research courses content and its applicability to their professional field. A semantic analysis found that university students tend to consider psychology as a science, but less than natural sciences. Moreover, the term "psychology" and "science" were semantically linked by concepts related to research (Richardson and Lacroix, 2021). Additionally, undergraduate psychology students tend to be more interested in practitioner activities than in scientific/research activities (Holmes, 2014).

Students report several factors that dissuade them from doing research; these include considering that research activities are time-consuming, difficulties associated with the lack of mentorship and funding (AlGhamdi et al., 2014; Siemens et al., 2010). Instructors of research methods classes often report that students have negative attitudes and disinterest in such courses (Gurung and Stoa, 2020). In part, attitudes towards research can be explained by variables such as research anxiety, the perceived importance and usefulness students attribute to research, and believing that research has an unbiased nature (Gredig and Bartelsen-Raemy, 2018). In this last regard, it is important to consider students' epistemic orientation.

Epistemic orientation refers to the individuals' preferences on how to gain and use knowledge (Silva Palma et al., 2018). One taxonomy of epistemic orientations identifies three main preferences (Royce, 1975; Silva Palma et al., 2018; Wilkinson and Migotsky, 1994): intuitive, rational, and empirical. The intuitive orientation assumes that knowledge is subjective and might be attained through metaphors and symbolisms. On the other hand, a rational orientation uses logic to evaluate arguments as true or false. An empiric orientation assumes that knowledge can only be attained through structured observations and experimentation. Science is greatly based on a combination of rational and empiric orientations.

Critical thinking is the process in which a person elaborates conclusions based on evidence (Wallmann and Hoover, 2012), focusing on argumentation and reasoning. This requires synthetic, introspective skills, skepticism, openness to new arguments or evidence, evaluating different options and their ramifications, dialogical thinking, self-questioning, self-monitoring, self-criticism (Garrett and Cutting, 2017; Reznitskaya and Sternberg, 2012; Sosu, 2013; Sternberg, 1987). Critical thinking is an essential element of scientific thinking (Shargel and Twiss, 2019), and an essential skill in the academic formation of psychologists. Consequently, students are, ideally, trained to admit the role of randomness, evaluate the methodological quality of arguments, understand the differences between correlation and causality, acknowledge the complex and multicausal nature of events, and understand the importance of falsification (Lawson, 1999; Lawson et al., 2015). Therefore it is evident that there is a link between critical thinking and research within the psychological sciences (Meltzoff and Cooper, 2018).

Recent studies have found that research and statistics courses may enhance students' knowledge of the topic without increasing their interest (Sizemore and Lewandowski, 2009). Specifically, teachers play an important role in developing students' research competencies, including its attitudinal component (Udompong et al., 2014). Students' satisfaction with university courses is related to teaching quality and expertise (Green et al., 2015). As such, it is vital to determine the role satisfaction with research courses plays in students' attitudes towards research.

The National Autonomous University of Honduras (UNAH) offers psychology programs in undergraduate (BA) and postgraduate (master's) degrees. The undergraduate program consists of 45 courses, of which 4 are mandatory-sequential Research Methods classes (UNAH, 2019). By the end of the degree, students are expected to be competent in elaborating research proposals, literature reviews, the basic design of quantitative and qualitative instruments, applying descriptive and basic inferential statistics, and writing technical reports. On the other hand, the postgraduate degree has 18 compulsory courses, of which 4 are mandatory research classes (UNAH, 2021). Their content is thesis-oriented, as it is a graduation requirement for the postgraduate programs of the UNAH.

Considering this, the purpose of our exploratory study was to test the following hypothesis: attitudes towards research are related to epistemic orientation, critical thinking, and satisfaction with research courses in psychology university students of Honduras. This while controlling for respondents' gender, current academic degree (undergraduate or postgraduate), number of research methods courses completed, number of research projects completed, and academic score.

## 2 Methods

### 2.1 Participants

The current study included students in the final year of their bachelor's degree, and students enrolled in a master's degree psychology program at a public university in Honduras. The sample was collected online through a non-probabilistic approach using volunteer and snowball sampling. Due to the COVID-19 pandemic, all university courses are held exclusively online. Considering this, invitations to participate in the study were sent via email to all 603 undergraduate students coursing final year classes and internships. Similarly, emails invitations were sent to all 62 masters' degree students. However, due to low response rates, students who completed the survey were also asked to send the email invitation to fellow students.

This resulted in a final sample size of 137 participants, of which 75.91% (*n* = 104) were undergraduate students, accounting for 17.24% of the population of undergraduate students. On the other hand, 24.09% (*n* = 33) were enrolled in a master's degree, representing 53.22% of the postgraduate population. Most respondents (*n* = 113; 82.48%) were female, while male students only accounted for 17.52% of the total sample (*n* = 24). The gender distribution in the sample is coherent with the population's demographic characteristics, in which 76.24% are female students, and 23.76% are male (National Autonomous University of Honduras, 2021).

The mean academic score was 83.75% (*SD* = 7.11); this represents the weighted average from all academic courses completed by the students. Students had completed an average of 5.08 research courses (*SD* = 1.96) and participated in an average of 4.18 research projects (*SD* = 2.86). The overall age of the respondents was 28.20 years (*SD* = 7.61). Specifically, undergraduate students had a mean age of 26 years (*SD* = 5.46), while master's degree students had a mean age of 35.12 years (*SD* = 9.23).

### 2.2 Variables and measures

#### 2.2.1 Attitudes towards research

Data was collected using the Attitudes Towards Research Scale-Revised (EACIN-R) (Aldana de Becerra et al., 2020), a revised version of the original EACIN (Aldana de Becerra et al., 2016). It consists of 28 items, with a five-point Likert-type response set, with scores varying from 1 *(completely disagree)* to 5 *(completely agree),* with higher scores indicating more favorable attitudes towards research. Some items included in the EACIN-R are: "All professionals should know how to do research", "I do not believe research should be taught at universities" and "I am interested in doing research activities". As measured by Cronbach's Alpha, the internal consistency for this sample was 0.89, 95% CI [0.86; 0.91].

#### 2.2.2 Epistemic orientation

The Epistemic Orientation Short Scale (EOSS) consists of 11 items with a five-point Likert-type response set, with scores varying from 1 *(completely disagree)* to 5 *(completely agree),* with higher scores indicating a more prevalent epistemic orientation. The EOSS measures the following subscales: rationalism (*α =* 0.71), intuitivism (*α =* 0.77), and empiricism (*α =* 0.72) (Silva Palma et al., 2018). The current study determined the internal consistency coefficients for each dimension: rationalism, *α =* 0.83, 95% CI [0.78; 0.87]; intuitivism, *α =* 0.65, 95% CI [0.54; 0.73]; empiricism *α =* 0.64, 95% CI [0.52; 0.73]. Some items from the EOSS include: "My opinions are commonly based on feelings and intuitions" (intuitivism), "I tend to make decisions based on reasons I can clearly explain" (rationalism), and "I tend to make decisions based on my experiences and practical situations" (empiricism).

#### 2.2.3 Critical thinking

The Critical Thinking Disposition Scale (CTDS) is an 11-item instrument with a five-point Likert-type response set, with scores varying from 1 *(completely disagree)* to 5 *(completely agree),* with higher scores indicating higher self-reported critical thinking disposition. The CTDS has a bi-dimensional structure consisting of two factors: critical openness and reflective skepticism. Previous research reported an overall Cronbach's alpha of 0.81 (Sosu, 2013), similar to the one found in the current study, *α =* 0.86, 95% CI [0.82; 0.89]. Some items included in the CTDS are: "I sometimes find a good argument that challenges some of my firmly held beliefs" (Critical Openness) and "I usually check the credibility of the source of information before making judgments" (reflective skepticism).

#### 2.2.4 Satisfaction with University Research Courses

The authors of the current study elaborated the Satisfaction with University Research Courses Scale (SURCS). Items were built by the authors and later sent to three Research Methods university professors who revised the wording and validity of every item. The experts rated each question on a 5-point scale according to their importance, pertinence, and wording; items with low scores were rephrased according to the experts' opinions. The final version of the SURCS consists of 12 Likert-type items with a five-point response set, with scores varying from 1 *(completely disagree)* to 5 *(completely agree)*. Higher scores indicate higher satisfaction with research university courses. The items reflects course content-related satisfaction, teacher satisfaction, perceived importance of the Research Methods courses, and personal satisfaction with such courses.

The instrument had an overall internal reliability of 0.91, 95% CI [0.89; 0.93], the average inter-item correlation was of 0.48, 95% CI [0.41; 0.54], Table 1 details the reliability for each item included in the SRUCS. Some of the items included in the SURCS are: "I enjoyed taking the Research Methods courses", "I believe my teachers of Research Methods courses had plenty experience as researchers", "I believe the content of the Research Methods courses is relevant".

**Table 1:** Item reliability for the satisfaction with University Research Courses Scale.

Item	Cronbach's *α*	Item-rest correlation
1. I enjoyed taking the Research Methods courses.	0.90	0.66
2. I believe the teachers of my Research Methods courses had plenty of scientific experience.	0.91	0.64
3. I believe the teachers of my Research Methods courses had good teaching strategies.	0.90	0.74
4. I enjoyed attending my Research Methods lectures.	0.90	0.63
5. My Research Methods courses were boring.	0.91	0.55
6. My Research Methods courses were interesting.	0.90	0.77
7. I learned a lot in my Research Methods courses.	0.90	0.75
8. I am satisfied with what I learned in my Research Methods courses.	0.90	0.72
9. My Research Methods courses had updated contents.	0.91	0.56
10. I believe the contents of my Research Methods courses are important.	0.91	0.54
11. I believe the contents of my Research Methods courses have helped me throughout my career.	0.91	0.60
12. I am satisfied with the teachers that instructed me in my Research Methods courses.	0.90	0.75

*Note.* Item 5 was inversely recoded.

#### 2.2.5 Demographic and educational questionnaire

Additional demographic and educational data were collected through a questionnaire that gathered information regarding respondents' gender (0 = male, 1 = female), age, current academic degree (0 = undergraduate, 1 = postgraduate), number of research methods courses completed, number of research projects completed, and self-reported academic grade. On this last point, students were asked to enter the academic grade as reported in their official university online certification. The academic grade is a score that ranges between 0 and 100.

### 2.3 Data analysis

Items were averaged to determine the total for each scale. An exploratory correlational analysis, using Pearson's *r*, was used to assess inter-variable dynamics. Comparisons between undergraduate and postgraduate students were made by using Student's t-test, a power analysis with its corresponding confidence intervals was also made. Later, a hierarchical linear regression model was used to explain the scores students achieved at the Attitudes Towards Research Scale-Revised (EACIN-R). The independent variables tested included: EOSS-rational, EOSS-intuitive, EOSS-empiric, CTDS-critical openness, CTDS-reflexive skepticism, and satisfaction with research courses. This while controlling for: gender, current academic degree, number of research methods courses completed, number of research projects completed, and academic grade. A post-hoc analysis was used to determine the achieved power of the regression model. An *α* = 0.05 was used as a significance threshold. Participants were required to answer all items; therefore, no missing data were included in the study. All statistical analyses were made using JASP (JASP Team, 2020).

### 2.4 Ethical considerations

The study design and execution were approved by the Ethical Committee of the Universidad Internacional Iberoamericana (UNINI), under certificate N˚ CE-025. All potential participants were presented with an Informed Consent form that included the study's purpose, confidentiality agreement, voluntary participation clause, data management, etc. Agreeing to the Informed Consent was required to allow students to participate in the study.

## 3 Results

Results indicate that students had an average score of 3.87 (*SD* = 0.50) in the Attitudes Towards Research Scale-Revised. The mean of the Satisfaction with Research University Courses Scale was 4.04 (*SD* = 0.71). The most prevalent epistemic orientation was the EOSS-Empiric disposition (*M* = 4.09; *SD* = 0.65), followed by EOSS-Rational (*M* = 3.87; *SD* = 0.73), and EOSS-Intuitive as less prevalent disposition (*M* = 3.40; *SD* = 0.75). Regarding critical thinking, CTDS-Reflexive-Skepticism scores (*M* = 4.31; *SD* = 0.69) were higher than CTDS-Critical Openness (*M* = 4.19; *SD* = 0.54).

Satisfaction with research courses and attitudes towards research were significantly higher for postgraduate students than for undergraduate respondents. Such differences are not only statistically significant (*p* < 0.01), but also achieve medium effect sizes (*d* = -0.64). Empiric and rational epistemic orientations are similarly scored by undergraduate and postgraduate students (*p* > .05); however, intuitive orientation is significantly lower for postgraduate respondents (*p* = 0.04). Critical thinking disposition subscales do not vary significantly between undergraduate and postgraduate students (*p* > .05). Table 2 provides a detailed description of mean differences, significance, and effect size.

**Table 2:** Score comparisons between undergraduate and postgraduate students.

Variable	Group	Mean	*SD*	*t*	*p*	Cohen's *d*	95% CI for Cohen's *d*	
							LL	UL
Satisfaction with research courses	Undergraduate	3.93	0.69	-3.18	<0.01	-0.64	-1.03	-0.24
	Postgraduate	4.37	0.70					
Attitudes towards research	Undergraduate	3.80	0.48	-3.20	<0.01	-0.64	-1.04	-0.24
	Postgraduate	4.11	0.50					
EOSS-Empiric	Undergraduate	4.05	0.67	-1.42	0.16	-0.28	-0.68	0.11
	Postgraduate	4.23	0.59					
EOSS- Intuitive	Undergraduate	3.47	0.77	2.05	0.04	0.41	0.01	0.80
	Postgraduate	3.17	0.60					
EOSS- Rationalism	Undergraduate	3.83	0.71	-1.12	0.26	-0.22	-0.62	0.17
	Postgraduate	3.99	0.77					
CTDS- Critical Openness	Undergraduate	4.16	0.57	-1.27	0.20	-0.25	-0.65	0.14
	Postgraduate	4.30	0.43					
CTDS- Reflexive Skepticism	Undergraduate	4.27	0.75	-1.27	0.20	-0.25	-0.65	0.14
	Postgraduate	4.45	0.44					

*Note. df* = 135.

A relational analysis determined that academic score is significantly and positively correlated (*p* < 0.05) with CTDS-Critical Openness, CTDS Reflexive Skepticism, EOSS-Empiric, EOSS-Rational, satisfaction with research courses, and attitudes towards research. On the other hand, EOSS-Intuitive is inversely related to academic scores and the number of research courses completed. The number of research projects completed was significantly and positively associated with CTDS-Reflexive Skepticism, EOSS-Rational, satisfaction with research courses, and attitudes towards research. Additionally, both rational and empiric orientations correlate positively with critical thinking domains. Attitudes towards research also have positive relationships with EOSS-Rational and EOSS Empiric, but are inversely related with EOSS-Intuitive, see Table 3.

**Table 3:** Correlational analysis between educational variables, critical thinking, and epistemic orientation.

Variable	Statistic	1	2	3	4	5	6	7	8	9
1. Number of research courses completed	*r*	—								
	*p*	—								
2. Number of research projects completed	*r*	**.46**	—							
	*p*	<.001	—							
3. Academic score	*r*	.14	.12	—						
	*p*	.10	.18	—						
4. CTDS-Critical Openness	*r*	-.06	.03	**.21**	—					
	*p*	.49	.72	.02	—					
5. CTDS-Reflexive Skepticism	*r*	.08	**.17**	**.25**	**.69**	—				
	*p*	.35	.04	<.01	<.001	—				
6. EOSS-Empiric	*r*	.01	.10	.13	**.40**	**.49**	—			
	*p*	.89	.24	.12	<.001	<.001	—			
7. EOSS-Intuitive	*r*	**-.19**	-.13	**-.25**	.01	-.11	.17	—		
	*p*	.02	.12	<.01	.88	.19	.05	—		
8. EOSS-Rational	*r*	.14	**.17**	**.28**	**.55**	**.46**	**.46**	-.03	—	
	*p*	.09	.04	<.01	<.001	<.001	<.001	.74	—	
9. Satisfaction with research courses	*r*	.14	**.21**	**.32**	**.28**	**.33**	**.36**	-.09	**.41**	—
	*p*	.09	.02	<.001	<.001	<.001	<.001	.29	<.001	—
10. Attitudes towards research	*r*	**.24**	**.25**	**.34**	**.41**	**.40**	**.45**	**-.18**	**.47**	**.51**
	*p*	<.001	<.001	<.001	<.001	<.001	<.001	.04	<.001	<.001

*Note.* Correlation coefficients were calculated through Pearson's *r*. Significant *p*-values (<0.05) are presented in bold letters.

Furthermore, a hierarchical regression model was used to determine how attitudes towards research are explained by critical thinking, epistemic orientation, and satisfaction with research courses. The base model, containing control variables, had an *r*^*2*^ of .197, *F* (5, 131) = 6.411, *p* < .001. The final model, containing all independent variables, had an *r*^*2*^ of .474, *F* (11, 125) = 10.229, *p* < 0.001, this constitutes a large effect size (Cohen, 1992), *f*^*2*^ = .901, with a high power >0.99. The changes between the base and final model are statistically significant, *r*^*2*^*Δ* = .277, *FΔ* = 3.818, *p* < 0.001.

While controlling for the academic degree, number of research courses completed, number of completed research projects, academic grade and gender, the following independent variables had a significant effect on attitudes towards research: satisfaction with research courses (*β* = 0.256, *p* = 0.001), empiric epistemic orientation (*β* = 0.254, *p* = 0.003), intuitive epistemic orientation (*β* = -0.149, *p* = 0.039) and critical openness (*β* = 0.197, *p* = 0.049). Rational epistemic orientation (*β* = 088, *p* = 0.32) and skeptic reflexiveness (*β* = -0.043, *p* = 0.665) yielded non-significant coefficients (*p* > 0.05), see Table 4.

**Table 4:** Regression model explaining students' attitudes towards research.

Model		Unstandardized Coefficients		Standardized *β*	*t*	*P*	*β* 95.0% CI	
		*β*	SE				LL	UL
1	(Constant)	2.417	0.57		4.238	0	1.289	3.546
	Academic degree	0.101	0.109	0.086	0.928	0.355	-0.115	0.318
	Number of research courses completed	0.03	0.023	0.117	1.314	0.191	-0.015	0.075
	Number of research projects completed	0.022	0.016	0.128	1.426	0.156	-0.009	0.054
	Academic score	0.016	0.007	0.226	2.415	**0.017**	0.003	0.029
	Gender	-0.195	0.108	-0.147	-1.796	0.075	-0.409	0.02
2	(Constant)	1,276	0.587		2.172	0.032	0.113	2,438
	Academic degree	0.05	0.093	0.043	0.539	0.591	-0.134	0.233
	Number of research courses completed	0.033	0.019	0.128	1.694	0.093	-0.006	0.072
	Number of research projects completed	0.008	0.013	0.048	0.631	0.529	-0.018	0.035
	Academic score	0.005	0.006	0.065	0.787	0.433	-0.007	0.016
	Gender	-0.099	0.093	-0.075	-1.070	0.287	-0.283	0.084
	Satisfaction with research courses	0.182	0.054	0.256	3.353	**0.001**	0.075	0.289
	EOSS-Empiric	0.197	0.065	0.254	3.047	**0.003**	0.069	0.326
	EOSS-Intuitive	-0.101	0.048	-0.149	-2.081	**0.039**	-0.196	-0.005
	EOSS-Rational	0.061	0.062	0.088	0.998	0.320	-0.06	0.183
	CTDS-Critical Openness	0.185	0.093	0.197	1.984	**0.049**	0.01	0.369
	CTDS-Reflexive Skepticism	-0.031	0.073	-0.043	-0.433	0.665	-0.175	0.112

*Note.* Significant *p*-values (<0.05) are presented in bold letters. All Variance Inflation Factors (VIF) scores range from 1.09 to 2.35, indicating no collinearity issues.

## 4 Discussion

The current research provides evidence that suggests that attitudes towards research are positively and significantly affected by students' satisfaction with research courses, empiric epistemic orientation, and critical openness. On the other hand, an intuitive epistemic orientation has significant detrimental effects on attitudes towards research. Students with high scores in critical thinking domains and empiric and rational dispositions, tend to achieve higher academic grades. Rationality and reflexive skepticism were related to the number of research projects completed by the student. While an intuitive disposition is inversely related to academic scores and the number of research courses completed.

Considering this, our study indicates that students' attitudes towards research could improve by reinforcing the quality of research methods courses, promoting empirical epistemic values and critical openness. On the first topic, knowledge of research methods is a premise of scientific thinking; therefore, effective research training should promote scientific thinking skills while considering students' epistemic beliefs (Murtonen and Salmento, 2019). Teaching students how to evaluate the credibility and validity of information sources is a key component to promote critical thinking (Carlson, 1995). Teachers should also promote inquiry-based activities in their classes; these include: students creating and answering their own questions, reciprocal peer questioning and, including questions that require holistic-integrative responses (King, 1995). Such methods should enhance critical thinking and rational epistemic orientation.

Defining questions and hypotheses, critical thinking, and epistemic understanding are vital to overcoming intuitive-based decisions and non-scientific beliefs, leading to an evidence-based approach to problem-solving (Murtonen and Salmento, 2019). An empiric epistemic orientation has significant effects on attitude towards research. Empiricism is highly driven by observational and experimental reports (American Psychological Association, 2020), and is an essential pillar of scientific research.

Our study provides evidence that an intuitive epistemic orientation has detrimental effects on students' attitudes towards scientific research. This finding is coherent with previous research made in a sample of psychotherapists, in which intuitive thinking was related to negative attitudes towards research, as well as more resistance to adopting evidence-based treatments in their professional practice. Psychotherapists with higher intuitive thinking were more willing to endorse alternative therapies and misconceptions about health (Gaudiano et al., 2011).

Likewise, critical openness was found to be a significant predictor of students' attitudes towards research. Considering that critical openness refers to the willingness to explore new or alternative arguments (Sosu, 2013), it is logical that such openness was a significant predictor of students' attitudes towards research. In this sense, prior research has determined that scientists, in contrast to non-scientists, report significantly higher scores on openness (Sato, 2016). Contemplating and evaluating new or alternative arguments is a key component to promote scientific development, and as such, these skills should be promoted in higher education settings. Teachers play an important part in enhancing students' critical thinking skills, playing a facilitator role, emphasizing the analytical process related to decision making, promoting discussion among peers, autonomous learning, and dialogical thinking (Reznitskaya and Sternberg, 2012; Sternberg, 1987).

Our findings indicate that the number of research courses completed by the students does not influence their attitudes towards research. This finding is coherent with Sizemore and Lewandowsk (2009), who concluded that completing research and statistics courses may enhance students' knowledge on the topic, without necessarily increasing their interest. Therefore, to better understand students' attitudes towards research, the focus should not reside on the number of research courses completed by the students, but rather on their satisfaction with such classes.

Satisfaction with research courses plays an important role in developing students' attitudes towards research. Thus, such courses should be taught by teachers highly trained in research and teaching skills, with updated, relevant, and applicable content that captures students' interest in research methods. This suggestion is in line with previous research, which identifies that teaching quality and expertise promote students' satisfaction with research courses (Green et al., 2015). In this sense, teacher engagement has significant effects on student engagement (Cardwell, 2011).

Overall, teachers should explicitly state and evidence the relationship between scientific thinking and research skills, as well as their application beyond academic activities. Students should also have clarity about the research process and what is expected of them as researchers. In this sense, quality feedback, adequate mentorship, peer support, and collaborative learning may enhance favorable attitudes towards the research process (Balloo, 2019).

Future studies should consider using qualitative and mixed methods designs to understand students' epistemic beliefs better, further exploring the meaning of psychology as a science. On the other hand, additional studies could specifically focus on postgraduate students and their attitudes and experiences on research activities, such as thesis writing.

The present study is not without limitations. The non-probabilistic selection process and the limited sample size may restrict the representativeness of the results. The nature of the epistemic, scientific, and attitudinal variables also possess an issue because it requires the respondents to have acquired a certain level of epistemic maturity (Murtonen and Salmento, 2019). Such awareness and metacognitive capabilities might not be adequately developed in all students. Additionally, the relatively low reliability of the EOSS subscales of Intuitivism (*α =* 0.65) and Empiricism (*α =* 0.64) is a limitation to consider when interpreting our research results. Future studies should also investigate further the psychometric properties of the SURCS. Finally, high scores in the EACIN-R indicate favorable attitudes towards research, and low scores indicate unfavorable attitudes. However, the EACIN-R lacks a system to categorize attitudinal scores through cut-off values (Aldana de Becerra et al., 2020). In this sense, more research is yet needed to further validate the scale in university populations.

## Declarations

### Author contribution statement

Miguel Landa-Blanco and Antonio Cortés-Ramos: Conceived and designed the experiments; Performed the experiments; Analyzed and interpreted the data; Contributed reagents, materials, analysis tools or data; Wrote the paper.

### Funding statement

This research did not receive any specific grant from funding agencies in the public, commercial, or not-for-profit sectors.

### Data availability statement

Data will be made available on request.

### Declaration of interests statement

The authors declare no conflict of interest.

### Additional information

No additional information is available for this paper.
